# Surgical Treatment of Idiopathic Macular Hole Using Different Types of Tamponades and Different Postoperative Positioning Regimens

**DOI:** 10.1155/2020/8858317

**Published:** 2020-12-03

**Authors:** M. Veith, J. Vránová, J. Němčanský, J. Studnička, M. Penčák, Z. Straňák, P. Mojžíš, P. Studený, D. P. Piñero

**Affiliations:** ^1^Department of Ophthalmology, University Hospital Kralovske Vinohrady and Third Faculty of Medicine, Charles University in Prague, Prague, Czech Republic; ^2^Department of Medical Biophysics and Medical Informatics, Third Faculty of Medicine, Charles University in Prague, Prague, Czech Republic; ^3^Department of Ophthalmology, University Hospital and Faculty of Medicine Ostrava, Ostrava-Poruba, Czech Republic; ^4^Department of Ophthalmology, University Hospital Hradec Kralové and Faculty of Medicine, Hradec Kralové, Charles University in Hradec Kralové, Czech Republic; ^5^Premium Clinic Teplice, Teplice, Czech Republic; ^6^Department of Optics, Pharmacology and Anatomy, University of Alicante, Alicante, Spain

## Abstract

**Purpose:**

To compare the effect of different types of intraocular tamponade and different types of postoperative positioning on the closure of idiopathic macular hole (IMH).

**Methods:**

Prospective randomized clinical trial enrolling 104 eyes of 100 patients (age, 57–87 years) undergoing MH surgery. All patients were operated on by an experienced surgeon using 25-gauge pars plana vitrectomy (PPV) and internal limiting membrane (ILM) peeling. Patients were randomized according to the type of intraocular tamponade and postoperative positioning into the following four groups: SF6 + nonsupine reading position (*n* = 26) (group 1), air + nonsupine reading position (*n* = 25) (group 2), air + prone position (*n* = 26) (group 3), or SF6 + prone position (*n* = 27) (group 4). The follow-up period was 6 months.

**Results:**

MH closure was achieved in 87 eyes (83.7 %) in the overall sample after the first surgery, with closure rates of 100%, 56%, 84.6%, and 92.6% in groups 1, 2, 3, and 4, respectively. The group 2 was significantly less successful compared to the other three groups (*p* < 0.05). MH of sizes ≤400 *µ*m was closed in 97.2% of cases after the first surgery, with no significant differences between groups (*p* = 0.219). MH with sizes over 400 *µ*m was closed in 70.9% of cases after the first surgery, with both groups with air tamponade being significantly less successful than group 1. The nonsupine reading position was subjected to a better subjective evaluation in terms of postoperative comfort and quality of sleep, with no differences between air and SF6 tamponade tolerance.

**Conclusion:**

PPV with ILM peeling, intraocular tamponade, and positioning remains the basic surgical approach in the treatment of IMH. For MH ≤ 400 *µ*m, a high closure rate can be achieved by combining air tamponade and nonsupine reading position. For macular holes >400 *µ*m, the greatest anatomical success can be achieved by using the SF6 tamponade in combination with the nonsupine reading position.

## 1. Introduction

The macular hole (MH) represents a defect of the fovea center in its full thickness from the internal limiting membrane (ILM) to the outer segments of photoreceptors. This pathological condition is characterized by painless decline in visual acuity, metamorphopsia, and central scotoma [1]. The idiopathic macular hole (IMH) is the most common presentation, being considered as incurable until 1991, when Kelly and Wendel published their successful results in 52 patients (58% of holes closed) after vitrectomy surgery and gas tamponade [2]. During the following years, the surgical procedure underwent a number of modifications in order to increase its success and safety.

Perioperative removal of ILM around the macular hole to increase the anatomical and functional success of the MH surgery was first described by Eckardt et al. in 1997 [3]. Subsequently, different authors confirmed the importance of ILM peeling for MH closure [4, 5]. Although the ILM peeling increases the anatomical and functional success of the MH surgery, it can also negatively affect the function and structure of the retina [6]. After the surgical release of the posterior vitreous, vitrectomy, and subsequent ILM peeling, the infusion fluid is routinely exchanged for air and subsequently for expansion gas [2]. The duration of the gas charge varies depending on the gas used and its concentration, ranging from 2 to 11 weeks (2–2.5 weeks for SF6, 4–6 weeks for C2F6, and 8–11 weeks for C3F8) [7]. A prone position is traditionally recommended for patients undergoing this surgical procedure, which is very uncomfortable and can cause some complications, such as back pain, sinusitis, or paralysis of the ulnar nerve [8]. The most optimal duration for this recommended positioning remains unclear and is very variable among published studies. The most recommended option is to maintain the prone position at least 8 hours a day for at least 5–7 days in order to maximize the contact of the bubble with the macular landscape [7, 9].

There is a close relationship between the type of tamponade chosen and the postoperative patient positioning regimen, to ensure that the tamponade bridges the MH. Even in the upright position, the gas bubble still bridges the hole, provided that the gas fills more than 50% of the vitreous space. Furthermore, long-lasting gas tampons fill more than 50% of the vitreous space in a longer period than short-acting gases or air. However, it is still unclear how long the gas must bridge the MH to close it. The initial size of the MH is the most relevant risk factor for the surgical success, being this apparently a key factor in the choice of the tamponade and the need for specific postoperative patient positioning [10]. The aim of this work was to compare the effect of combinations of different types of intraocular tamponades (air or SF6 gas) and different types of postoperative patient positioning on IHM closure (prone or nonsupine reading position). This article is of high clinical relevance although new advances have suggested that postoperative positioning may be not mandatory for MH closure considering that new techniques of hole closure seem to adequately relieve the tangential traction and some even to provide a scaffold for possible regeneration. However, it should be considered that these techniques, such as the ILM flap technique, are used by some surgeons primarily for large MH, not routinely for all MH.

## 2. Methods

### 2.1. Patients

This prospective, randomized, clinical series enrolled a total of 104 eyes with IMH of 100 patients with ages ranging from 57 to 87 years that underwent MH surgery at the Department of Ophthalmology of the University Hospital Kralovske Vinohrady in Prague (Czech Republic). All patients were informed about the nature and risks of the study and signed a written informed consent to be enrolled in it according to the tenets of the Declaration of Helsinki. Likewise, the protocol of the study was approved by the ethics committee of the Royal Vinohrady University Hospital on March 1, 2016. Inclusion criteria were diagnosis of IMH stage 2 to 4 according to Gass [1] and signing informed consent. Exclusion criteria included the following conditions: previous pars plana vitrectomy (PPV), eye injury, myopia ≥6 diopters, any intraocular or periocular infection or active intraocular inflammation (infectious conjunctivitis, keratitis, scleritis, endophthalmitis, infectious blepharitis, or uveitis) in the evaluated eye on the day of surgery and other macular diseases that could affect the surgical outcome (wet form of age-related macular degeneration, central serous chorioretinopathy, macular telangiectasia, diabetic macular edema, or edema in retinal vein occlusion).

**Figure 1:**
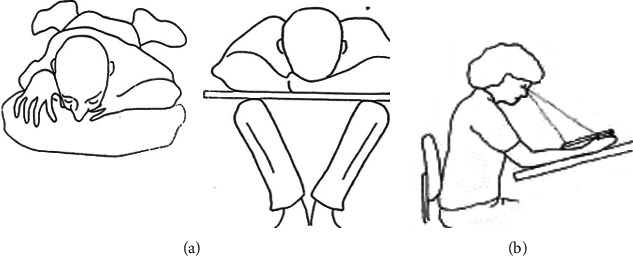
Drawing scheme showing the configuration of the two types of positioning recommended in the current study: (a) prone position; (b) nonsupine reading position.

Patients enrolled in the study were divided into 4 groups according to the intraocular tamponade used in the MH surgery and the type of patient positioning recommended in the postoperative period using a randomization generator (https://www.sealedenvelope.com/simple-randomiser/v1/lists):

  Group 1: SF6 tamponade + nonsupine reading position (Figure 1)
  Group 2: tamponade air + nonsupine reading position
  Group 3: tamponade air + prone position (Figure 1)
  Group 4: SF6 tamponade + prone position

The assignment to the relevant group was performed just before the start of the surgical procedure in the operating room.

### 2.2. Clinical Examinations

In all patients, a complete preoperative examination was performed including anterior segment slit lamp examination, including biomicroscopy of the posterior segment of the eye under artificial mydriasis, air tonometry, measurement of uncorrected (UCVA) and best-corrected visual acuity (BCVA) using the ETDRS (Early Treatment Diabetic Retinopathy Study) optotypes in decimal values, manifest refraction, and analysis of the retinal structure confirming the diagnosis of MH by spectral domain optical coherence tomography (OCT) (Spectralis OCT, Heidelberg Engineering, Heidelberg, Germany). The first postoperative examination was performed the day after surgery, with additional postoperative examinations at 1, 3, and 6 months after surgery. The same spectrum of examinations as before surgery was also performed on all these postoperative examinations, with the exception of the first examination in which no OCT examination was included. If there was significant cataract development or progression in the postoperative period, patients underwent standard phacoemulsification cataract surgery with implantation of a posterior chamber intraocular lens.

### 2.3. Surgical Procedure

All patients were operated on by the same experienced surgeon (MV) using the 25-gauge PPV of the Constellation surgical system (Alcon, Fort Worth, TX, USA). The area around the eye and the conjunctival sac was disinfected with a 5% povidone-iodine solution, the operating field was covered with a sterile drape, and a dilator was placed. After oblique transconjunctival insertion of trocars through the pars plana at 3.5–4 mm from the limbus, vitrectomy was initiated. In the case of fixation of the ZSM to the posterior pole of the eye, it was released by suction of the vitrectomy approximately at the equator region. ILM peeling was performed using Eckardt forceps and a contact macular lens. Brilliant blue (Ocublue Plus, Aurolab) was used to facilitate the identification of the membranes for their safe and complete removal. The ILM peeling area covered approximately 2 PD (papilla diameter). Afterwards, a complete exchange of fluid for air and, if necessary, instillation of expansive gas 20% SF6 was performed following the protocol defined according to the preoperative randomization. After extraction of the trocars, the tightness of the sclerotomies was checked, and in case of leakage, they were sutured with Vicryl 8–0 absorbable sutures. All surgeries were performed under retrobulbar anesthesia (3 ml of Marcain + 2 ml of Supracain).

After surgery, the following recommendations were given to patients depending on the group of randomization assigned (Figure 1):

  Groups 3 and 4: to keep the head as much as possible in the prone position for 3 days, with the greatest emphasis on maintaining this position for the first 24 hours after surgery
  Groups 1 and 2: to keep looking during the daily activities as if reading for 3 days, not laying on the back at night (recommendation of sleeping on the stomach or side at night)

Furthermore, each patient also received a questionnaire with three questions to evaluate the severity of the recommended postoperative regimen.

### 2.4. Statistical Analysis

Statistica version 9 from Statsoft was used for statistical analysis. Normality of quantitative data samples was evaluated using the Kolmogorov–Smirnov test in order to confirm if parametric or nonparametric statistical tests had to be used. Contingency table analysis and Pearson's chi-square test were used to determine the difference in macular hole closure success rates between the four groups of patients evaluated and also between pairs of groups. Fisher's exact test was used in the case of the comparison of small sample sizes. A paired Student's *t*-test (Wilcoxon test if the sample was not normally distributed) was used to compare the visual acuity before and after surgery for the whole sample as well as for each group separately. The unpaired Student's *t*-test (Mann–Whitney test if the sample was not normally distributed) was used to compare postoperative intraocular pressure values between individual operated groups. A *p* value of less than 0.05 was considered as statistically significant.

## 3. Results

A total of 104 eyes of 100 patients (76 women and 24 men) were included in the study. Both eyes were operated in two women and two men. Mean age of patients was 70.8 years (range, 57 to 87 years). A total of 74 eyes were phakic at the time of IMH diagnosis, whereas 30 eyes were pseudophakic. The preoperative mean value of decimal BCVA was 0.15 in the whole sample, with a range of variation from 0.05 to 0.50. The average size of the MH at its narrowest point in the hole sample was 408.5 *µ*m (range, 133 to 741 um). In 44 eyes (42.3%), an epiretinal membrane (ERM) was also present. In 89 eyes (85.6%), the posterior vitreous membrane was attached, being then necessary to release it perioperatively. The main characteristics of the patients enrolled in each individual group are summarized in Table 1. The follow-up period was 6 months.

### 3.1. MH Closure Rate Analysis

Full MH closure was achieved in 87 eyes (83.7%) from the whole group after the first surgery. The MH closure success rates after primary surgery in each group are displayed in Table 2. A flattening and complete closure of the edges of the MH was considered as an anatomical success. The group with air tamponade and nonsupine reading position (group 2) was statistically significantly less successful compared to the other three groups (group 2 vs. 1, *p* < 0.001; 2 vs. 3, *p* = 0.025; 2 vs. 4, *p* = 0.003). The differences in the MH closure success rates between the other three groups (1, 3, and 4) were not statistically significant (group 1 vs. 3, *p* = 0.110; 1 vs. 4, *p* = 0.255; and 3 vs. 4, *p* = 0.316). MH with a size of ≤400 *µ*m closed after the first surgery in 97.2% of cases, with no statistically significant difference in closure success rates between the individual-operated groups (*p* = 0.219). Macular holes of more than 400 *µ*m in size closed in 70.9% of cases after the first operation. Both groups with air tamponade (2 and 3) were statistically significantly less successful compared to the SF6 + nonsupine reading position group (group 1) (1 vs. 2, *p* < 0.001; 1 vs. 3, *p* = 0.037). No statistically significant differences in the closure success rates were found between the two groups with air tamponade (*p* = 0.081), as well as between the two groups with gas tamponade SF6 (*p* = 0.120).

A second PPV with gas tamponade due to a not complete closure of the MH after primary PPV was needed in 16 eyes. Only in 1 eye, a third PPV with a silicone oil tamponade was required. Furthermore, one patient with an open MH refused a second operation after primary PPV. In the last follow-up visit, the MH was closed in a total of 103 eyes (99.0%).

### 3.2. Visual Outcome Analysis

At the end of the follow-up period, mean decimal BCVA in the whole group improved to 0.56 (range from 0.16 to 1.0) (*p* < 0.001). BCVA worsened in one eye (patient number 9 from group 4, worsening by 2 ETDRS lines) and remained at the preoperative level in another eye. In the rest of cases, BCVA improved (98.1%). BCVA improved by 3 ETDRS lines or more in 92 eyes (88.5%). At the end of the follow-up period, 15 of the 17 eyes (88.2%) not achieving a MH closure after primary surgery also improved by more than 3 ETDRS lines. At the last postoperative visit, no significant differences in the level of BCVA between individual were found (*p* > 0.05).

### 3.3. Questionnaire Outcomes

Concerning the first question performed (was the postoperative positioning uncomfortable for you?), patients rated the prone position statistically significantly worse than the nonsupine reading position (*p* = 0.010). In the second question (did you experience impaired sleep quality due to positioning?), patients with the recommendation of the prone position reported a more significant impairment of sleep quality than patients with the recommendation of the nonsupine reading position (*p* = 0.001). Regarding the third question (did the air/gas tamponade bother you with its duration?), patients rated air tamponade as significantly worse (*p* = 0.028).

### 3.4. Complications

All sclerotomies were sufficiently sealed, not being necessary to suture them. On the first postoperative day, mean IOP was 16.0 mm Hg (range, 6 to 40 mm Hg) in the overall sample, not observing hypotension below 6 mm Hg in any operated eye. Mean IOP values on the first postoperative day were 18.6, 12.5, 14.9, and 17.9 mm Hg in groups 1, 2, 3 and 4, respectively. Differences in IOP the first day after surgery were statistically significant among groups 1 and 2 (*p* < 0.001), 1 and 3 (*p* = 0.011), 2 and 4 (*p* < 0.001), and 3 and 4 (*p* = 0.035). In the postoperative period, IOP ≥25 mm Hg was observed in 4 eyes (3.8%) that were quickly resolved with local antiglaucoma therapy.

The frequency of complications was relatively low. Small retinal tears at the end of the procedure during the peripheral retinal examination were detected in 10 cases (9.6%). Likewise, peripheral vitreoretinal traction and malignant degeneration were observed in 20 eyes (19.2%) that were successfully treated with laser photocoagulation or cryopexy. Cataract surgery was performed in 28 of 74 phakic eyes (37.8%) due to a progress in the opacification of the crystalline lens after MH surgery. No other postoperative complications, such as retinal detachment or endophthalmitis, were recorded in this cohort.

## 4. Discussion

Many authors have confirmed the relevance of ILM peeling for a successful closure of MH, considering as a standard step in MH surgery. In 1997, Eckardt et al. reported a successful closure of MH in 92% of cases using this surgical procedure [3]. Subsequently, many other authors have confirmed the positive effect of ILM peeling on MH closure. Kwok et al. [11] reported an anatomical success rate of the operation of 89% in patients with MH grades 3 and 4, in which ILM peeling was performed compared to a rate of 59% in patients operated on without ILM peeling. In another study enrolling 127 patients, Lois et al. [4] detected a closure of MH in 84% of undergoing surgery with ILM peeling, whereas in eyes undergoing surgery without ILM peeling, the MH closure was only achieved in 48% of cases (*p* < 0.001). In the current study, ILM peeling was performed in all patients. Although ILM peeling has been shown to rise the anatomical success of the MH surgical procedure, it should be also mentioned that the ILM peeling can also lead to defects of the retinal architecture, atrophy of the macular area (especially in the temporal part), and significant defects of retinal sensitivity when measured with microperimetry [12–14].

Besides ILM peeling, the type of intraocular tamponade chosen as well as the mode of postoperative patient positioning have a fundamental influence on the closure of the MH. Kelly and Wendel's surgical procedure using a gas tamponade and subsequent face-down patient positioning has become a standard in the treatment of MH [2]. Several studies have demonstrated the excellent effect of gas tamponade in combination with patient face-down positioning on the closure of MH. Almeida et al. [15] found that MH (stages 1–3) was successfully closed with SF6 tamponade and patient pronation position for three days in 49 of 50 eyes (98%) [15]. Our research group published in 2015 a study showing the results of MH surgery using either SF6 or C3F8 as gas tamponade and recommending a prone position for three days to all patients [16]. In this previous series, a successful outcome was obtained after the first operation in 92.5% of eyes [16]. In the current series, a similar MH closure rate (92.6%) was found in the SF6 + pronation group. The effect of long-term gas tamponade using C3F8 and C2F6 is similar to that found for a shorter tamponade using SF6. In 2008, a comparison of the effect of different types of gas tamponade in combination with the patient prone position was performed, confirming that 90% and 91% of MH were closed using SF6 and C3F8, respectively (*p* = 0.91). [17] Modi et al. [18] also compared the effect of SF6 and C3F8, not detecting a statistically significant difference in the effect of both gases and obtaining MH closure rates of 86.4% and 86.5% using SF6 and C3F8, respectively (*p* = 0.98). However, a lower incidence of cataract and postoperative ocular hypertension were observed with the use of SF6.

The gas bubble contributes to the closure of the MH by several mechanisms, being essential for the maintenance of a dry macula and its isolation from vitreous fluid [19, 20]. The duration of the gas tamponade varies depending on the gas used and its concentration, ranging from 2 to 11 weeks [7]. During this period, the patient loses the binocular vision, with the impossibility of driving or travelling by plane. Approximately 60% of patients rate gas tamponade as uncomfortable or very uncomfortable [21]. Gas tamponade also accelerates the development and progression of cataracts [22]. In contrast, air is absorbed more rapidly in the eye, with an acceleration of the recovery and the return of the patient to normal life. In our cohort, patients were asked by means of a questionnaire to evaluate the duration of the tamponade, with no more favorable perception of the air tamponade compared to SF6. In phakic eyes, the half-life of air is 1.3 days [23]. In pseudophakic eyes, the air filling of the eye is larger, with an average time of complete air absorption of 10 days [24]. When a pronation position is recommended, the air bubble keeps the macula sufficiently isolated from the vitreous fluid, and therefore, excellent surgical results can be achieved. Sato and Isomae [25] reported a MH closure rate of 91.3% after surgery with ILM peeling in patients in which the prone position was recommended for one day during air tamponade. Hejsek et al. [26] reported a MH closure rate of 93.1% using air tamponade. Hasegawa et al. [27] achieved a similar MH closure rate (92.3%) in a group of eyes with air tamponade, whereas the MH closure rate was 90.1% in another group of eyes with SF6 tamponade (the difference did not reach statistical significance, *p* = 0.132). Similarly, Usui et al. [28] compared the effect of air tamponade and SF6 in MH with sizes up to 500 *µ*m, being successful in 100% of cases in both groups, but using a significantly shorter positioning time in the air tamponade group. In another study, similar success rates were also reported, with a closure of 30 from a sample of 33 MHs after 3 days of positioning with air (90.9%) [29]. In our series including 57.7% of MH with sizes ≥ 400 *µ*m, 84.6% of MH was successfully closed in the group of eyes using air tamponade and prone position, with no significant differences compared to the group of eyes using SF6 tamponade and prone position (*p* = 0.316).

The success of the MH closure when using prone positioning was very high, even regardless of the type of the tamponade used. However, many patients referred that the maintenance prone positioning was difficult or very difficult [30]. It should be considered that the MH typically develops in elderly patients that commonly have physical obstacles, such as obesity or spinal problems to maintain this position. The prone position represents a significant source of discomfort for the patient. In some patients, the prone position may be also the cause of some complications, such as ulnar nerve palsy or ulnar pressure ulcer [8]. For this reason, the need for a prone position is still currently a matter of debate. In our cohort, patients perceived the nonsupine reading position significantly better than the prone position in terms of both comfort and sleep quality. Many authors have confirmed that a high anatomical success can be also achieved with other modes of patient positioning (nonsupine reading position). Iezzi and Kapoor [31] reported the results of MH surgery using ILM peeling (8000 *µ*m wide), SF6 tamponade, and reading position for 3–5 days in 68 eyes showed a successful MH closure in 100% of the eyes. In another study, a MH closure was obtained in 203 of 204 eyes (99.5%) undergoing surgery with ILM peeling, SF6 tamponade, and recommendation to patients of maintaining a reading position [32]. Other authors reported nonsignificant differences in the MH closure success rate using gas tamponade between recommending to patients postoperatively the reading position or the pronation position (91.2%–97.1%) [21, 33, 34]. These authors did not observe a difference between reading and pronation position groups, even for MH larger than 400 *µ*m. In our series, the MH was closed in all cases after the first operation in the group of eyes in which the SF6 tamponade was used and the patient nonsupine reading position was recommended.

Forsaa and Krohn [24] used a combination of reading position and air tamponade in an attempt to increase the postoperative comfort. In the group of MH ≤ 400 *µ*m, a successful closure was reached in 95% of cases, whereas only a successful closure was achieved in 57% of eyes in the group of MH > 400 *µ*m. This study only enrolled pseudophakic eyes in which a larger air filling can be achieved, with sufficient coverage of the MH and an efficient isolation from vitreous fluid. It should be considered that the air bubble occupies an average of 59% of the vitreous space on the first postoperative day, which seems to be sufficient to close MH with sizes up to 400 *µ*m. Previously published study groups have shown that up to 96% of MH closes within the first 24 hours [29, 31, 35]. On the third postoperative day, only an average of 39% of air is present in the vitreous space [24]. MH of more than 400 *µ*m therefore seems to require a longer isolation time from vitreous fluid to heal than smaller MH, which is in line with the observations of other authors [29, 36]. In the group of eyes of the current series using air tamponade and patient nonsupine reading position, MH closure was only achieved in 56% of eyes, being a significantly worse result than those obtained in the air + prone position group and in the SF6 gas + nonsupine reading position. However, MH closure was achieved in 88.9% of eyes with MH with sizes up to 400 *µ*m, which is worse outcome than that obtained by Forsa and Krohn [37]. Two main factors may have accounted for this: the inclusion of phakic eyes in our sample and a better postoperative cooperation of patients in the study of Forsa and Krohn [37] in which the tennis ball technique was used to eliminate the supine position.

Finally, it should be acknowledged that air tamponade is also safer than gas due to the possible elevation of IOP in the postoperative period. After PPV with gas tamponade, up to 35.6% of patients may have IOP above 30 mm Hg [38]. In a previous study of our research group, 28.5% of patients with detached retina and treated with PPV and gas tamponade had IOP ≥ 25 mmHg on the first postoperative day group of patients [39]. In contrast, eyes with air tamponade have shown the lowest risk of IOP elevation (cumulative risk of 11.5% for IOP elevation ≥ 30 mmHg after 48 hours) [40]. In our series, the mean values of IOP on the first postoperative day in the groups using air tamponade were 12.5 and 14.9 mmHg, whereas mean values of 18.6 and 17.9 mm Hg were found in the group of eyes using SF6 tamponade, being the differences statistically significant. According to this, the use of air tamponade should be especially considered in patients with preexisting glaucoma.

In conclusion, PPV with ILM peeling, intraocular tamponade, and positioning remains the basic surgical approach in the treatment of IMH. The type of tamponade and positioning should be chosen based on the size of the macular hole, the condition of the crystalline lens, and the overall condition of the patient. For MH ≤ 400 *µ*m, their closure can be achieved with high success by combining an air tamponade with a patient's nonsupine reading position, especially in pseudophakic eyes. For MH > 400 *µ*m, the greatest anatomical success can be achieved using SF6 gas tamponade in combination with the nonsupine reading position. Patients tolerated the nonsupine reading position better than the prone position. The duration of the SF6 tamponade versus the shorter air tamponade does not seem to be perceived as a benefit by patients.

## Figures and Tables

**Table 1:** Preoperative characteristics of the operated patients in each individual group.

	Group 1 (SF6 + nonsupine reading position)	Group 2 (air + nonsupine reading position)	Group 3 (air + prone position)	Group 4 (SF6 + prone position)
Number of eyes	26	25	26	27
Mean age (range) (years)	70.2 (63–80)	71.3 (57–86)	69.2 (59–79)	72.4 (61–87)
Mean decimal BCVA (range)	0.11 (0.05–0.33)	0.10 (0.05–0.33)	0.19 (0.05–0.50)	0.18 (0.01–0.50)
Mean IMH size (range) (*µ*m)	426.7 (178–612)	446.4 (178–711)	405.4 (148–741)	358.7 (133–652)
Number of phakic eyes (%)	16 eyes (61.5)	20 eyes (80.0)	21 eyes (80.8)	17 eyes (63.0)
Eyes with ERM (%)	9 eyes (34.6)	10 eyes (40.0)	15 eyes (57.7)	10 eyes (37.0)
Eyes with posterior vitreous membrane attached (%)	24 eyes (92.3)	20 eyes (80.0)	20 eyes (76.9)	25 eyes (92.6)

BCVA, best-corrected visual acuity; IMH, idiopathic macular hole; ERM, epiretinal membrane.

**Table 2:** Macular hole closure success rates after primary surgery in each individual group.

	Group 1 (SF6 + nonsupine reading position) (%)	Group 2 (air + nonsupine reading position) (%)	Group 3 (air + prone position) (%)	Group 4 (SF6 + prone position) (%)
MH closure success of whole sample	100	56.0	84.6	92.6
MH closure success of IMH ≤ 400 *µ*m	100	88.9	100	100
Successful closure of IMH > 400 *µ*m	100	37.5	71.4	77.8

IMH, idiopathic macular hole.

## Data Availability

The data used to support the findings of this study are available upon request to the first author.
